# Biophysical modeling of excitability and membrane integration at the single cell and network levels

**DOI:** 10.1186/1471-2202-12-S1-P218

**Published:** 2011-07-18

**Authors:** Marco Arieli Herrera-Valdez, Adrian Smith, Maytee Cruz-Aponte, Erin C McKiernan

**Affiliations:** 1 Mathematical, Computational, and Modeling Sciences Center, Arizona State University, Tempe, AZ 85287, USA; 2 School of Evolution and Social Change, Arizona State University, Tempe, AZ 85287, USA; 3 School of Life Sciences, Arizona State University, Tempe, AZ 85287, USA

## 

Ion channels facilitate the diffusion of specific ions across neuronal membranes. If large enough, this movement of charge creates currents that may change the membrane potential. Biophysical models of membrane potential assume the trans-membrane currents flow within an “equivalent electrical circuit” in which ion channels are represented by resistors arranged in parallel. The functions representing the trans-membrane currents mediated by channels are typically written using Ohm's law. It is possible to describe channel-mediated currents by taking diffusion into consideration [1], but such formulations are not widely used in the literature. Here we present a model of membrane potential in which channel gating and current density are derived from first principles of thermodynamics, assuming that currents are produced by electrodiffusion. These models display properties that cannot be observed in conductance-based models, such as rectification of membrane currents. Guidelines for parameter estimation, and specific rules to adjust the model against experimental data, are presented along with examples of parameter regimes that yield representations of specific electrophysiological signatures with a biophysically sound baseline. Bifurcation analysis is used to describe transitions between qualitatively different behaviors of the model and link them to functionally relevant properties observable in neurons of different types. Network extensions are constructed using realistic synaptic input and local field potential oscillations to illustrate how networks may display potentially different responses to afferent input depending on the intrinsic properties of the participating neurons. The electrodiffusion formulation presented here constitutes a theoretical improvement over conductance-based models that may advance our current understanding of dynamical behavior in single cells and networks.
